# Pulse oximetry findings in newborns with antenatally diagnosed congenital heart disease

**DOI:** 10.1007/s00431-018-3093-2

**Published:** 2018-02-05

**Authors:** Isabel E. Mawson, Pratusha L. Babu, John M. Simpson, Grenville F. Fox

**Affiliations:** 1 Department of Neonatology, Neonatal Intensive Care Unit, Evelina London Children’s Hospital, St. Thomas’ Hospital, Westminster Bridge Road, London, SE1 7EH UK; 2 Department of Cardiology, Evelina London Children’s Hospital, St. Thomas’ Hospital, Westminster Bridge Road, London, SE1 7EH UK; 3 Department of Women and Children’s Health, King’s College London, St.Thomas Hospital, Westminster Bridge Road, London, SE1 7EH UK

**Keywords:** Neonate, Congenital abnormality, Oximetry, Postnatal care, Neonatal screening

## Abstract

A retrospective review of admission preductal oxygen saturations of neonates with antenatally diagnosed critical congenital heart disease (CCHD) was performed to investigate the differences in newborn pulse oximetry (Pulsox) by specific CCHD diagnosis. Saturations were recorded at median of < 1 h (range < 1–9 h) after delivery. Data was stratified by CCHD diagnosis and analysed according to the three different admission Pulsox thresholds, ≤ 90, ≤ 92 and ≤ 95%. Of the 276 neonates studied, 208 were clinically well at admission, with no co-morbidities, gestation > 34 weeks and birth weight > 1.8 kg. A statistically significant increase in the proportion with low admission saturations was seen using ≤ 95% saturation threshold (72% (95% CI 66–78)) compared to ≤ 92% (52% (95% CI 46–59)) and ≤ 90% (46% (95% CI 39–52)). Sub-group analysis found the proportion of neonates with low saturations varied according to the specific CCHD diagnosis with only 20–42% of neonates with aortic stenosis, coarctation of the aorta and pulmonary stenosis having saturations ≤ 95%.

*Conclusion*: The proportion of neonates with low admission oxygen saturation varied by CCHD diagnosis with those without critically reduced pulmonary blood flow not having low admission saturations, in general, even using the ≤ 95% threshold which had the highest proportions of abnormal saturations. This data may assist developing Pulsox screening policies.

**Table:** 

**What is Known:** • *The addition of pulse oximetry (Pulsox) screening to the routine newborn examination increases the sensitivity of CCHD detection. Pulsox screening is also highly specific for CCHD in asymptomatic neonates, with low false-positive rates.* • *Early diagnosis of CCHD improves patient outcomes in relation to both morbidity and mortality.*
**What is New:** • *The proportion of affected infants with an abnormal Pulsox result varies by CCHD diagnosis and screening threshold. In our study using the ≤ 95% threshold gave the highest proportion of neonates with abnormal saturations at admission.* • *In general, Pulsox yield of abnormal results is low for CCHD diagnoses not associated with critically reduced pulmonary blood flow; however, increasing the Pulsox threshold increased the proportion of infants with an abnormal result.*

**Electronic supplementary material:**

The online version of this article (10.1007/s00431-018-3093-2) contains supplementary material, which is available to authorized users.

## Introduction

Identifying newborn infants with critical congenital heart disease (CCHD) before they suffer cardiovascular collapse is of paramount importance in optimizing outcome following surgical or catheter intervention [8, 13, 14]. Data related to antenatal identification of specific congenital heart disease diagnosis has suggested a benefit in terms of outcome for transposition of the great arteries (TGA) [3], hypoplastic left heart syndrome (HLHS) [24] and coarctation of the aorta (CoA) [9]. However, national antenatal screening programs for congenital heart disease have shown that this form of screening is imperfect [4]. Pulse oximetry (Pulsox) screening of all newborns is being considered for national implementation in the UK with the aim of detecting affected infants at an early stage, particularly those in whom pulmonary or systemic blood flow will be duct-dependent [25].

Although there are many previously published studies on Pulsox screening, they have all included relatively low numbers of infants with CCHD [23]. Until now, therefore, it has been difficult to demonstrate whether Pulsox screening is likely to be equally effective at identifying all types of CCHD.

We hypothesised that the proportion of neonates with antenatally diagnosed CCHD with low oxygen saturations at admission would be influenced by the specific diagnosis of congenital heart disease.

Our hospital is a tertiary paediatric cardiac centre, which is co-located with a large tertiary maternity service and neonatal intensive care facilities, in a region where a high proportion of CCHD is antenatally detected. This, coupled with a policy of routinely recording oxygen saturations on admission to the neonatal unit, provided an opportunity to gauge the potential detection of different types of known congenital heart disease according to different thresholds of oxygen saturation.

## Methods

A retrospective review of admission preductal oxygen saturation levels in newborn infants was conducted on a cohort of infants born with antenatally diagnosed CCHD in one tertiary cardiac neonatal unit between January 2010 and May 2014. During this time, it was routine practice for the neonatal team to attend all deliveries of infants with known CCHD, followed by routine neonatal unit admission. The admission protocol for all infants included measurement of preductal (right hand) oxygen saturations. Preductal oxygen saturations were measured using a Masimo SET LNCS® Neonatal Pulse Oximeter Adhesive Sensor and IntelliVue Module (Masimo, Irvine, CA, USA). Standardised training amongst nursing staff ensured all preductal saturations were measured appropriately.

Two databases, (Heartsuite (Systeria, Glasgow UK) and Badgernet (Clevermed, Edinburgh UK)), were searched in order to identify our cohort. Inclusion criteria: all fetuses/neonates with antenatally diagnosed CCHD following referral of the mother to the fetal cardiology and fetal medicine service at our hospital, and later born at the same hospital, with CCHD being confirmed postnatally. Exclusion criteria: infants transferred after delivery at another hospital and postnatal diagnoses of CCHD were excluded from analysis. For consistency with previous work, we have adopted the definition of CCHD from a recently published meta-analysis of Pulsox screening [23], including hypoplastic left heart syndrome (HLHS), pulmonary atresia (PA)––with duct-dependent pulmonary flow including those with or without intact ventricular septum and MAPCA’s, critical/severe pulmonary valve stenosis (PS), critical aortic stenosis (AS), interruption of the aortic arch (IAA), coarctation of the aorta (CoA), tetralogy of Fallot (ToF), transposition of the great arteries (TGA) and total anomalous pulmonary venous drainage (TAPVD).

Data collected included demographics, specific CCHD diagnosis, co-morbidities, admission preductal oxygen saturation and requirement for ventilation and/or supplemental oxygen. All data were collated and statistically analysed using Microsoft Excel. The numbers and percentages of neonates with low admission saturations, using different saturation thresholds of ≤ 90, ≤ 92 and ≤ 95%, were calculated for the whole cohort and then individually for each specific CCHD diagnosis. Ninety-five percent confidence intervals were calculated for the percentage of neonates with low admission saturation. A one-tailed chi-squared test was performed to evaluate potential differences between the three admission saturation thresholds.

Data from neonates who were clinically well at admission and were only admitted to the neonatal unit due to antenatal diagnosis of CCHD (i.e. > 34 weeks gestation, ≥ 1.8 kg, with no co-morbidities and not requiring oxygen, ventilation, or prostaglandin E infusion at admission) were then selected and analysed separately as above. This selection was deliberated in order to represent neonates with no other reason for neonatal unit admission apart from antenatally diagnosed CCHD implying that they would have been cared for on the postnatal ward had an antenatal diagnosis not been known, and would therefore have been ideal for newborn Pulsox screening.

Ethics committee approval was not required as no additional intervention or data collection above routine care was carried out.

## Results

Two hundred seventy-six eligible neonates were identified. Two hundred sixty-six (96%) of these had admission oxygen saturations recorded in the database and were therefore included in data analysis. Median birth weight was 3.09 kg (range 0.67–4.83 kg) and median gestation 38^+4^ weeks (range 27^+5^–41^+5^). Tewnty-four (9%) were preterm (< 37 weeks gestational age). Table 1 shows the percentage of neonates with each CCHD diagnosis and demographics by diagnosis.

**Table 1:** Demographics by specific CCHD diagnosis

Diagnosis	*N* (%)	Gestational age (weeks)		Median birth weight (kg)
		Median	< 37/40 number (%)	
AS	5 (2)	38 + 5	0 (0)	2.93
CoA	53 (20)	38 + 3	7 (13)	2.89
HLHS	45 (17)	38 + 5	4 (8)	3.15
IAA	7 (3)	38 + 6	0 (0)	2.95
PA	16 (6)	38 + 4	1 (6)	2.95
PS	17 (6)	38 + 4	4 (24)	2.85
TAPVD	6 (2)	38 + 1	2 (33)	2.70
TGA	74 (28)	38 + 4	3 (4)	3.23
ToF	43 (16)	38 + 5	3 (7)	3.08
Total	266 (100)	38 + 4	24 (9)	3.09

Thirty-eight (14%) had co-morbidities including preterm delivery, respiratory distress syndrome, intra-uterine growth retardation (birth weight < 3rd centile) and other congenital anomalies. Twenty-two (8%) had respiratory co-morbidities at admission. Thirty-one (12%) were ventilated on admission, and 30 (11%) were requiring oxygen on admission (Table 2). None were receiving a prostaglandin E infusion at the time of admission and therefore at the time oxygen saturations were measured.

**Table 2:** Percentages of neonates requiring ventilation and/or oxygen at admission by CCHD diagnosis

Diagnosis (total *N*)	*N* (%) ventilated at admission	*N* (%) requiring oxygen at admission
AS (5)	0 (0)	0 (0)
CoA (53)	4 (8)	6 (11)
HLHS (45)	4 (9)	2 (4)
IAA (7)	2 (29)	1 (14)
PA (16)	3 (19)	3 (19)
PS (17)	3 (18)	3 (18)
TAPVD (6)	3 (50)	1 (17)
TGA (74)	8 (11)	8 (11)
ToF (43)	4 (9)	6 (14)
Total (266)	31 (12)	30 (11)

Two hundred sixty (98%) neonates had admission oxygen saturation recordings taken within 1 h of birth (range < 1–9 h). Forty-eight percent (95% CI 42–54%) of admission saturations were ≤ 90%, 56% (95% CI 51–63%) ≤ 92% and 76% (95% CI 71–81%) ≤ 95%. These correspond with the percentage of neonates in our cohort who would be identified as potentially having CCHD by pulse oximetry screening at each of the above thresholds.

A further analysis was performed on those with no extra-cardiac anomalies, who were born ≥ 34-week gestation age, were ≥ 1.8 kg and were clinically well at birth. This represents a selected cohort who might have been well enough to avoid admission to the neonatal unit had there not been an antenatal diagnosis of CCHD. Two hundred eight infants (78%) met these criteria: 46% (95% CI 39–53%) of admission saturations were ≤ 90%, 52% (95% CI 46–60%) ≤ 92% and 72% (95% CI 66–78%) ≤ 95%. For this group, an admission saturation threshold of ≤ 95% was statistically and significantly more sensitive than either ≤ 90% (*P* < 0.0001) or ≤ 92% (*P* < 0.0001), with no statistically significant difference between the ≤ 92 and ≤90% thresholds (*P* > 0.1). Similar results were found for the whole cohort. In addition, when considering the whole cohort, there was also a statistically significant increase in the number of neonates with low saturations using the ≤ 92% threshold compared to ≤ 90%. Tables 3 and 4 show the number and percentage of infants with admission saturations below each threshold by specific diagnosis for those with no other co-morbidities (Table 3) and the whole cohort (Table 4), as well as detailing which threshold comparisons yielded statistically significant increases in infants with low saturations. There were statistically significant increases between the percentage of neonates with low admission saturations, without co-morbidities, by threshold for CoA (*P* < 0.01 for ≤ 95% compared to ≤ 90% and *P* < 0.05 for ≤ 95% compared to ≤ 92%), HLHS (*P* < 0.0001 for ≤ 95% compared to ≤ 90% and *P* < 0.0005 for ≤ 95% compared to ≤ 92%), and ToF (*P* < 0.005 for ≤ 95% compared to ≤ 90% and *P* < 0.05 for ≤ 95% compared to ≤ 92%). Similar results were also found for the whole cohort.

**Table 3:** Number and percentage of neonates with low admission saturation by CCHD diagnosis, excluding neonates < 1.8 kg, < 34 weeks gestation, with co-morbidities, ventilatory and/or oxygen requirement

Diagnosis (*N*)	Admission pulse oximetry oxygen saturation threshold (%)					
	≤ 90		≤ 92		≤ 95	
	*N*	% (95% CI)	*N*	% (95% CI)	*N*	% (95% CI)
AS (5)	0	0 (0–0)	0	0 (0–0)	1	20 (0–55)
CoA (41) ^**,***^	6	15 (3–26)	8	20 (7–32)	17	42 (26–57)
HLHS (38) ^**,***^	14	37 (22–52)	19	50 (34–66)	34	90 (80–99)
IAA (4)	1	25 (0–67)	1	25 (0–67)	3	75 (33–100)
PA (12)	8	67 (40–93)	10	83 (62–100)	11	92 (76–100)
PS (11)	3	27 (1–54)	4	36 (8–65)	4	36 (8–65)
TAPVD (3)	3	100 (100–100)	3	100 (100–100)	3	100 (100–100)
TGA (62)	52	84 (75–93)	53	86 (77–94)	55	89 (81–97)
ToF (32) ^**,***^	9	28 (13–44)	11	34 (20–51)	21	66 (49–82)
Total (208) ^**,***^	96	46 (39–53)	109	52 (46–59)	149	72 (66–78)

(*N* number with oxygen saturation below threshold)

^*^Statistically significant increase in the number of infants with oxygen saturations ≤ 92% compared to ≤ 90% threshold

^**^Statistically significant increase in the number of infants with oxygen saturations ≤ 95% compared to ≤ 92% threshold

^***^Statistically significant increase in the number of infants with oxygen saturations ≤ 95% compared to ≤ 90% threshold

**Table 4:** Number and percentage of neonates with low admission saturation by CCHD diagnosis, including all neonates regardless of treatment requirement, gestational age, weight or co-morbidities

Diagnosis (*N*)	Admission pulse oximetry oxygen saturation threshold (%)					
	≤ 90		≤ 92		≤ 95	
	*N*	% (95% CI)	*N*	% (95% CI)	*N*	% (95% CI)
AS (5)	0	0 (0–0)	0	0 (0–0)	1	20 (0–55)
CoA (53)^**,***^	9	17 (7–27)	13	25 (13–36)	27	51 (37–64)
HLHS (45) ^**,***^	17	38 (24–52)	23	51 (37–66)	40	89 (80–98)
IAA (7)	3	43 (6–80)	3	43 (6–80)	5	71 (38–100)
PA (16) ^***^	10	63 (39–86)	13	81 (62–100)	15	94 (83–100)
PS (17)	5	29 (8–51)	8	47 (23–71)	9	53 (29–77)
TAPVD (6)	4	67 (29–100)	6	100 (100–100)	6	100 (100–100)
TGA (74)	64	87 (79–94)	65	88 (80–95)	68	92 (86–98)
ToF (43) ^**,***^	16	37 (23–52)	20	47 (32–61)	31	72 (59–86)
Total (266) ^*,**,***^	128	48 (42–54)	151	57 (51–63)	202	76 (71–81)

(*N* number with oxygen saturation below threshold)

^*^Statistically significant increase in the number of infants with oxygen saturations ≤ 92% compared to ≤ 90% threshold

^**^Statistically significant increase in the number of infants with oxygen saturations ≤ 95% compared to ≤ 92% threshold

^***^Statistically significant increase in the number of infants with oxygen saturations ≤ 95% compared to ≤ 90% threshold

As this study only includes preductal oxygen saturations, those lesions where preductal oxygen saturations are expected to be higher than postductal oxygen saturations can be expected to be under identified by our method. Table 5 in supplementary material shows the expected effect of the structural abnormality in each type of CCHD on preductal and postductal oxygen saturations along with the proportion of infants in this study with each type of CCHD who had admission saturations lower than each threshold.

## Discussion

Pulse oximetry has been extensively investigated to screen newborn infants for unrecognised critical congenital heart disease [23, 28], with almost 400,000 screened infants included in publications to date [1, 2, 6, 7, 10–12, 16–21, 28]. A major limitation of the published data is that the number of infants with CCHD in any individual study is small, given the incidence of congenital heart disease. The largest study published to date [28] included only 157 babies with CCHD, with small numbers for any specific CCHD diagnosis.

The setting of our unit, with colocation of tertiary neonatal and cardiac services provided an opportunity to retrospectively analyse pulse oximetry findings in a large cohort of infants in whom congenital heart disease had been diagnosed before birth. This enabled analysis by specific CCHD diagnosis using different oxygen saturation thresholds. It should be emphasised that our approach was not ‘screening’ because all infants had known congenital heart disease following prenatal diagnosis by fetal echocardiography.

We found that for neonates without co-morbidities, the sensitivity of preductal pulse oximetry to detect CCHD ranged from 46 to 72%.

The consensus Pulsox screening threshold for further investigation is < 95% [5, 26]. In our cohort, the sensitivity of pulse oximetry to detect CCHD was higher using ≤ 95% threshold compared to the two lower thresholds. However, this was not consistently observed for all cardiac lesions (Tables 3 and 4). Although the use of a higher threshold may increase the proportion of false-positives requiring investigation, previous studies have demonstrated, such false-positives may still benefit from prompt management (such as that leading to earlier diagnosis of neonates with sepsis and pneumonia [22]).

Even after removal of clinically unstable neonates, the proportion in the different Pulsox threshold groups varies according to the CCHD diagnosis (Tables 3 and 4). Even at the lowest pulse oximetry threshold of ≤ 90%, the majority of our cohort with PA, TGA, and TAPVD had abnormal values. The haemodynamics of these lesions are characterised by duct-dependent pulmonary blood flow (PA), failure of mixing of oxygenated and deoxygenated blood (TGA), or obligate right to left shunting of blood at atrial level (TAPVD).

A statistically significant increment in sensitivity of pulse oximetry for CoA, HLHS, and ToF was noted when the saturation threshold was increased from ≤ 90% or ≤ 92% to ≤ 95%. The data for CoA and HLHS is clinically important because the systemic arterial circulation is duct-dependent, and a benefit of presentation prior to onset of heart failure or circulatory collapse has been reported [9, 24]. In HLHS, there is an obligate left to right shunt at atrial level and the systemic arterial circulation is maintained by blood flow from the right ventricle into the pulmonary artery and right to left through the arterial duct. The systemic arterial oxygen saturations are equal in the upper and lower limbs and reflect the pulmonary to systemic flow ratio. Even with oxygen saturations measured early after birth in our study, only the highest threshold (≤ 95%) was associated with an abnormal result in the majority of cases. With the continued fall in pulmonary vascular resistance during the first days after birth, oxygen saturations increase so that the sensitivity of detection by pulse oximetry is also likely to fall. In coarctation of the aorta, in contrast to HLHS, there is antegrade flow of blood through the left heart, so that it would be expected for preductal saturations to be normal or near normal in the first hours after birth and even after the arterial duct begins to constrict. This is consistent with the results we present with a relatively poor sensitivity whatever the threshold used. The combination of measurement of both preductal and postductal saturation may be useful, but coarctation of the aorta was not detected in the majority of cases in a large Swedish study and depended for the most part on weak femoral pulses for detection [6].

The incremental change in pulse oximetry sensitivity for tetralogy of Fallot reflects the balance between right ventricular outflow tract obstruction and ductal flow from the aorta into the pulmonary circulation. There is no published data to support improved outcome following either prenatal or early detection prior to closure of the arterial duct, so the early detection of this lesion is probably less critical; however, we do recognise that cases with severe PS are duct-dependent and their early detection is necessary to avoid severe hypoxia upon duct closure; this is reflected in our centre’s practice advising in-house delivery for such patients.

Data from our study show that for certain CCHD lesions, AS, PS and CoA, even with the use of a higher saturation threshold (≤ 95%), the proportion of infants with an abnormal result was low (20, 36 and 42%, respectively). The lower sensitivity of pulse oximetry screening for CoA has previously been recognised by Valmari [27] and Meberg [15]. Valmari [27] also noted the lower sensitivity of pulse oximetry screening for PS.

## Limitations

The presentation of our data is complicated by the very fact that CCHD was known in these infants. Also, the results we present may not be strictly comparable to the results in published screening studies/screening protocols because measurement of oxygen saturation was within 1 h of birth rather than between 6 and 72 h and preductal rather than preductal and postductal [5, 26]. The arterial duct is likely to have been patent at this time, and this may also have affected the readings.

Our measurement of oxygen saturations much earlier than screening studies was done according to unit protocol and avoided inclusion of oxygen saturations recorded after commencing prostaglandin E to maintain ductal patency. However, despite the difference in timing of the investigation, the sensitivity of pulse oximetry readings using ≤ 95% threshold in our cohort was 72% (95% CI 66–78%), which is similar to that in a recent meta-analysis of pulse oximetry screening [23]. Many screening protocols disagree on the best timing for measurement of oxygen saturations [5, 26], in particular whether this is done on the first or second day. Due to the limitations of our study, larger and more comprehensive work is required to adequately answer this question.

Our study design was retrospective which enabled only preductal oxygen saturation data to be collected as these saturations were measured routinely on admission to the unit whereas postductal saturations were not. A recent multi-centre Norwegian study of early pulse oximetry [16] showed a 77% sensitivity to detect CCHD using postductal measurements at 1–21 h of age. Our results, using preductal measurements was similar to that paper (16). For some cardiac lesions, the haemodynamics mean that the preductal and postductal saturations will be equal. Such lesions include pulmonary atresia, critical pulmonary valve stenosis and tetralogy of Fallot which are characterised by obstruction to pulmonary blood flow so that ductal flow is left to right. Other lesions with equal preductal and postductal saturations include common mixing lesions such as total anomalous pulmonary venous drainage and hypoplastic left heart syndrome. In transposition of the great arteries, the postductal saturations are higher than preductal so that a higher sensitivity for detection will be achieved by measurement of preductal saturations. The forms of congenital heart disease where our omission of measurement of postductal saturations will impact on sensitivity are those where there is antegrade flow of blood out of the left ventricle, but where postductal flow is maintained by right to left flow through the arterial duct. This will include severe aortic valve stenosis, coarctation of the aorta and interruption of the aortic arch. For these lesions, the data we present will underestimate the sensitivity of pulse oximetry if postductal saturations had also been measured (see Table 5 supplementary material).

Repeat preductal and postductal saturation measurements after 24 h of age would also be likely to increase the sensitivity of Pulsox screening for duct-dependent lesions as the duct constricts. Ours was not a screening study but one of prenatally diagnosed CCHD so that it was impossible to follow individual infants through to day 2 without commencement of drugs necessary to maintain ductal patency.

In our study, the sensitivity of early measurement of preductal oxygen saturation for HLHS is relatively high; however, approximately 10% escape detection. It is likely that this group have an initially high pulmonary blood flow and non-restrictive communication at atrial level. Of note, this group of infants is at risk for rapid deterioration of the systemic blood flow with a further decrease of pulmonary vascular resistance and (early) constriction of the ductus arteriosus.

Our retrospective study design, including only infants with prenatally diagnosed CCHD and no control group, means we are unable to draw conclusions regarding the specificity of pulse oximetry at each of the threshold. A further limitation is the small absolute number of infants with some types of lesion, despite the large overall cohort with CCHD we have analysed. Having larger sample sizes would allow for meaningful analysis of corresponding echocardiographic data, particularly in drawing conclusions about profiles of specific forms of CCHD that may go undetected.

Our results suggest that pulse oximetry screening would be a useful adjunct to the national neonatal screening program to identify CCHD. However, ‘one size does not fit all’ in this situation and we must use pulse oximetry screening with caution and in conjunction with thorough clinical examination of the newborn to ensure that lesions with low sensitivity are still identified.

## Conclusion

In this study, with the largest reported cohort of CCHD diagnoses, oxygen saturations were recorded as abnormal in the majority of cases, even within the first hour of life. The highest proportions of abnormal results were observed with the highest oxygen saturation threshold (≤ 95%). Using any threshold, the proportion of abnormal results varied depending on CCHD diagnosis, reflecting lesion-specific haemodynamics.

## Electronic supplementary material

ESM 1 (DOCX 22 kb)

## Abbreviations

AS: Aortic stenosis
CCHD: Critical congenital heart disease
CoA: Coarctation of the aorta
HLHS: Hypoplastic left heart syndrome
IAA: Interrupted aortic arch
PA: Pulmonary atresia
PS: Pulmonary stenosis
Pulsox: Pulse oximetry
TAPVD: Total anomalous pulmonary venous drainage
TGA: Transposition of the great arteries
ToF: Tetralogy of Fallot
