# Supplementary material for: Pulse oximetry findings in newborns with antenatally diagnosed congenital heart disease
Source: Eur J Pediatr. 2018 Feb 5;177(5):683–9. doi: 10.1007/s00431-018-3093-2 (PMC5899118; doi:10.1007/s00431-018-3093-2)
Supplement: Supplementary file 1 — (DOCX 22 kb) [file 431_2018_3093_MOESM1_ESM.docx]

**Supplementary material**

Table 5: The expected physiological effect of each CCHD lesion on pre and postductal saturations.

| Diagnosis (Total No.) | Expected Oxygen Saturations | N with preductal oxygen saturation ≤90% (%) | N with preductal oxygen saturation ≤92% (%) | N with preductal oxygen saturation ≤95% (%) |
| --- | --- | --- | --- | --- |
| AS (5) | Preductal > post | 0 (0) | 0 (0) | 1 (20) |
| CoA (41) | Preductal > post | 6 (15) | 8 (20) | 17 (42) |
| HLHS (38) | Preductal = post | 14 (37) | 19 (50) | 34 (90) |
| IAA (4) | Preductal >post | 1 (25) | 1 (25) | 3 (75) |
| PA (12) | Preductal = post | 8 (67) | 10 (83) | 11 (92) |
| PS (11) | Preductal = post | 3 (27) | 4 (36) | 4 (36) |
| TAPVD (3) | Preductal = post | 3 (100) | 3 (100) | 3 (100) |
| TGA (62) | Preductal < Post | 52 (84) | 53 (85) | 55 (88) |
| ToF (32) | Preductal = Post | 9 (28) | 11 (34) | 21 (66) |
| Total (208) |  | 96 (46) | 109 (52) | 149 (72) |
